# Effects of behavioral patterns and network topology structures on Parrondo’s paradox

**DOI:** 10.1038/srep37028

**Published:** 2016-11-15

**Authors:** Ye Ye, Kang Hao Cheong, Yu-wan Cen, Neng-gang Xie

**Affiliations:** 1 Department of Mechanical Engineering, Anhui University of Technology, Anhui Ma’anshan 243002, China; 2 Engineering Cluster, Singapore Institute of Technology, 10 Dover Drive, Singapore 138683, Singapore

## Abstract

A multi-agent Parrondo’s model based on complex networks is used in the current study. For Parrondo’s game A, the individual interaction can be categorized into five types of behavioral patterns: the Matthew effect, harmony, cooperation, poor-competition-rich-cooperation and a random mode. The parameter space of Parrondo’s paradox pertaining to each behavioral pattern, and the gradual change of the parameter space from a two-dimensional lattice to a random network and from a random network to a scale-free network was analyzed. The simulation results suggest that the size of the region of the parameter space that elicits Parrondo’s paradox is positively correlated with the heterogeneity of the degree distribution of the network. For two distinct sets of probability parameters, the microcosmic reasons underlying the occurrence of the paradox under the scale-free network are elaborated. Common interaction mechanisms of the asymmetric structure of game B, behavioral patterns and network topology are also revealed.

In Parrondo’s paradox, playing two losing games in a random or periodic order results in a winning outcome^1^. The original example provided in ref. 1 involves playing two losing games, A and B. However, a player might eventually win by playing the two games in a random or periodic order^1^. Specifically, game A plays an “agitating” role whereas game B plays a “ratcheting” role. After game A is first played, the player’s capital changes, which could either result in a winning or losing state. This sets up an “agitation” in the ensuing games played. When game B is played next, several combinations of asymmetric branches of outcomes may appear depending on the outcome of game A. Some branches in game B are more favorable and it is these asymmetric structures that form a “ratcheting” mechanism, eventually leading to a net gain in capital.

Parrondo’s paradox has been demonstrated in both individual-player^1^ and group versions^2^ of game A. For the individual-player version, the capital of the individuals is changed, revealing the “agitating” role of game A. For the group version of game A, there are *N* participants involved. Game A is assumed to be a zero-sum game between two individuals which are chosen randomly. The “agitating” role of game A is now elicited through the interactions between any two selected individuals. The zero-sum game between individuals leads to the distribution of capital between them. In a multi-agent Parrondo’s model of a network carrier, Ye *et al*. proposed a structure, “link A” (e.g. network structure evolution) which provides the “agitating” role of game A in the *s*-player version^3^. Both the simulation and theoretical analysis have shown that the “agitating” role of the network evolution can produce the paradoxical effect in a multi-agent Parrondo’s model.

For game B, its “ratcheting” role depends on three aspects – the individual capital^1^,^2^,^3^,^4^, recent history of wins and losses^5^ and the spatial neighboring states^6^,^7^,^8^. The former two pertain to the individual-agent version of game B, whereas the third one pertains specifically to the multi-agent or group version. In ref. 6, Toral proposed a group version of game B that depended on the neighboring players. In particular, he specified an ensemble of *N* players, with each one occupying a certain space. Depending on the losing and winning states of each spatial neighboring environment, the outcome structure of game B would vary. In this multi-agent model, there are networks of one-dimensional line and two-dimensional lattice. For any player *i* of the one-dimensional line, the outcome structure of game B could be divided into four scenarios^7^ according to *i*’s left and right neighboring states of gain or loss. For the two-dimensional lattice, the outcome structure of game B could be divided into five scenarios^8^ according to *i*’s four neighboring states of gain or loss (up, down, left and right) (in this case, the positions of the winning and losing states of the neighbors are not distinguished).

For game B that depends on the spatial neighboring environment, Toral^6^ analyzed it on a one-dimensional line. Based on the mean-field theory, Toral concluded that only a “weak” Parrondo effect (two games combine to form a better-performing game) rather than a “strong” Parrondo effect (two losing games combine to form a winning game) could result. A “strong” Parrondo effect was not correctly predicted by the mean-field analysis but was observed from the numerical simulation. Based on the discrete-time Markov chain, Mihailovic *et al*. theoretically analyzed game B on one dimensional line^7^ and on a two-dimensional lattice^8^. Compared to the results based on the mean-field theory and the numerical simulation performed by Toral^6^, Mihailovic observed that the result based on the discrete-time Markov chain was more accurate than the one based on the mean-field theory and closer to the result based on the numerical simulation. In order to perform a theoretical analysis for large *N*, Ethier *et al*.^9^,^10^ proposed a state-dimension reduction method. By simultaneously considering the group version of game A proposed by Toral in ref. 2 and the group version of game B in ref. 6, Xie *et al*.^11^ proposed a multi-agent Parrondo’s model and analyzed each game and a randomized combination of the games using the discrete-time Markov chain. Furthermore, the corresponding transition probability matrix, stationary probability distribution and expectation were obtained. The conditions under which the weak or strong paradox occurred were established with their corresponding parameter space.

Generally, the population occupies a certain network space. At present, the network carrier version of game B is only limited to the one-dimensional ring network and the two-dimensional lattice network, under which the neighboring number of the nodes is fixed (i.e. the neighboring environments of all nodes are isomorphic). Thus, it is easy to design and simulate the structure of game B. However, actual networks are usually complex, with many types of intricate topologies, such as random graphs, small-world networks and scale-free networks. An important difference between such complex networks and the two-dimensional lattice is that the node degree (i.e., the number of neighboring nodes) is not the same. Based on the above reasons, Ye *et al*.^12^ proposed a spatial dependence structure of game B applied to arbitrary topologies. The simulation results have confirmed that the size of the region of parameter space that elicits Parrondo’s paradox depends on the heterogeneity of the degree distributions of the networks. In particular, higher heterogeneity yielded a larger region of the parameter space where the strong paradox occurred.

Importantly, in ref. 12, the interaction between individuals in game A was designed on the basis of competition. Given the great variability in individual behaviors, an important question to ask is, do different competitive and cooperative behaviors in game A have an influence on the paradoxical effect? For the same behavioral patterns in different network carriers, how does the heterogeneity of the degree distributions of networks affect the parameter space where the paradox occurs? The simulations carried out in this paper set out to answer these questions.

## Model

**Figure 1:**
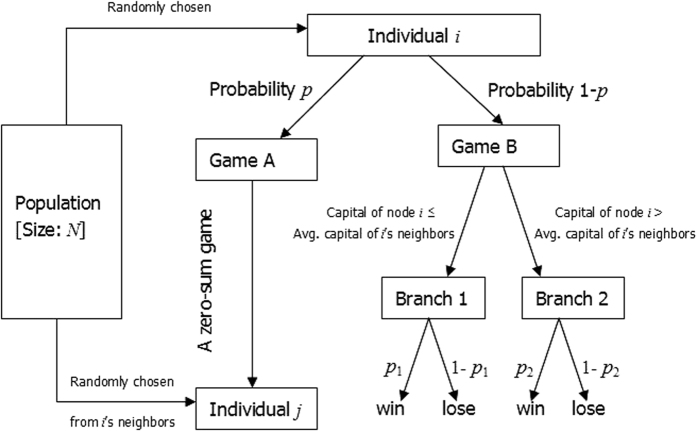
The multi-agent Parrondo’s model based on complex networks^12^.

The multi-agent Parrondo’s model based on complex networks is shown in Fig. 1^12^. The model consists of two games, A and B. Consider complex networks with *N* nodes^12^. The game modes include playing game A and game B individually and playing a randomized game A + B. The randomized game A + B means a probabilistic sequence of games A and B. The dynamic processes of the randomized game A + B are as follows: on each round of the game, one node ‘*i*’(the subject) is chosen at random from *N* nodes to play game A (with probability *p*) or game B (with probability 1–*p*). When game A is played, a node ‘*j*’ (the object) should be chosen at random from *i*’s neighbors (i.e. node *j* and node *i* are connected). The specific form of game A between node *i* and node *j* is determined by the interactive relationship between them.

### Zero-sum game between individuals – Game A

Designed as a zero-sum game, game A is used to represent the interactive mechanism between individuals. It has no impact on the total benefit of the population, but only changes the distribution of the benefit in the population. When node *i* wins, node *j* pays one unit to node *i*. That is, node *i*’s capital *C*_*i*_(*t*) increases by one unit, and node *j*’s capital *C*_*j*_(*t*) decreases by one unit; conversely, *i* pays one unit to *j*. That is, node *i*’s capital *C*_*i*_(*t*) decreases by one unit, and node *j*’s capital *C*_*j*_(*t*) increases by one unit. In this study, by considering different competitive and cooperative behaviors between individuals^13^, behavioral patterns are categorized into the following five types:

(1) The competitive pattern with a focus on competition (hereinafter referred to as the “Matthew effect”. The winning and losing probabilities are defined according to the current individual capital. The winning probability of node *i* at time *t* is determined according to Equation (1) below.


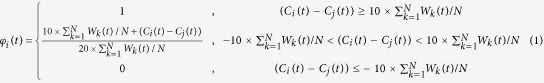


where: *φ*_*i*_(*t*) represents the winning probability of node *i* at time *t. W*_*k*_(*t*) = *C*_*k*_(*t*)−*C*_0_. *W*_*k*_(*t*) is the fitness of node *k* at time *t*, *C*_*k*_(*t*) denotes the capital of node *k* at time *t*, *C*_0_ is the initial capital of all individuals, and *N* is the population size.

(2) The cooperative pattern with a focus on harmony (hereinafter referred to as ‘Harmony’): the “rich” node pays the “poor” one, i.e., when *C*_*i*_(*t*) > *C*_*j*_(*t*), node *i* pays one unit to node *j*; when *C*_*i*_(*t*) ≤ *C*_*j*_(*t*), *j* pays one unit to *i*.

(3) The cooperative pattern: node *i* pays one unit to node *j* for free.

(4) Poor-competition-rich-cooperation mode (Hereinafter referred to as PCRC): when *C*_*i*_(*t*) ≤ *C*_*j*_(*t*), node *i* competes with node *j*. The winning probabilities of nodes *i* and *j* are 0.5, respectively. When *i* wins, *j* pays one unit to *i*; conversely, *i* pays one unit to *j*; when *C*_*i*_(*t*) > *C*_*j*_(*t*), node *i* cooperates with node *j*, then node *i* pays one unit to node *j* for free.

(5) The random pattern: node *i* randomly chooses a competitive or cooperative pattern to play game A. When the competitive mode is chosen, the winning probabilities of nodes *i* and *j* are 0.5, respectively. When *i* wins, *j* pays one unit to *i*; conversely, *i* pays one unit to *j*; when the cooperative mode is chosen, then node *i* pays one unit to node *j* for free.

### Game B

The structure of game B consists of two branches, which are generated according to the capital of node *i* and its neighbors (refer to Fig. 1). In branch 1, when the capital of node *i* is less than or equal to the average capital of all of its neighbors, the winning probability is *p*_1_. In branch 2, when the capital of node *i* is greater than the average capital of all of its neighbors, the winning probability is *p*_2_. Therefore, if we play game B with branch 1, a random number from 0 to 1 is generated randomly. Next, we compare the above random number with the winning probability of branch1, *p*_1_. If the random number is less than or equal to *p*_1_, the capital of node *i* increases by one unit. Otherwise, the capital of node *i* decreases by one unit. Branch 2 of game B may be deduced by analogy.

### Computer simulations and analysis

The fitness of any *i*_*th*_ individual at time *t* is defined as follows:


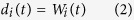


The average fitness of the population *d* at time *t* is


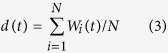


The condition under which the weak paradox occurs can be expressed by Equation (4):


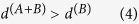


The condition under which the strong paradox occurs can be expressed by Equation (5):


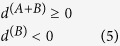


where: *d*^(*B*)^ and *d*^(*A*+*B*)^ represent the average fitness of the population of game B and the randomized game A + B respectively.

### The influence of the heterogeneity of the degree distributions on the paradoxical effect in different behavioral patterns

To analyze the influence of the network topology structures on the multi-agent Parrondo’s model, computer simulations based on complex networks were performed by implementing each of the above five behavioral patterns in game A. We hypothesized that the heterogeneity of the degree distribution of the network would have an effect on the strong and weak paradoxical results. To control the heterogeneity manipulation, we used the following two approaches. Firstly, in order to reflect the progressive changes from a two-dimensional lattice to a random graph, we start with a two-dimensional lattice and use the rewiring mechanism to generate a random graph^14^. The number of rewiring times, *L*, is the controlling index of the heterogeneity of the degree distribution. Secondly, to reflect the progressive changes from a random graph to a scale-free network, we use a model based on the degree distribution with adjustable parameters of the network and use the corresponding construction algorithm^15^. The parameter *α* ∈ [0, 1]is the controlling index of the heterogeneity of the degree distribution.

**Figure 2:**
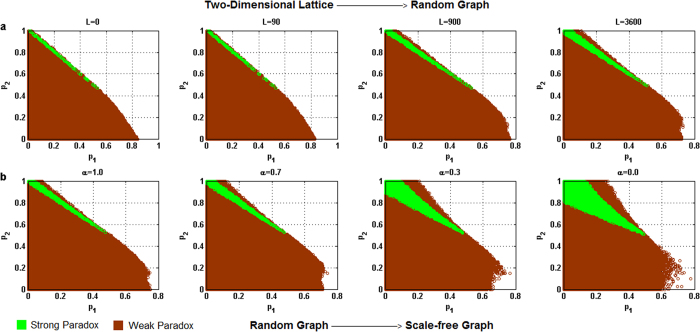
Influence of the heterogeneity of the degree distributions on the paradoxical effect in the Matthew mode. (**a**) The number of rewiring times *L* = 0, 90, 900 and 3,600, which corresponds to the networks changing gradually from a two-dimensional lattice to a random network. (**b**) The parameter α = 1.0, 0.7, 0.3 and 0.0 corresponds to the networks progressively changing from a random network to a scale-free network. The population size *N* is 900. The average degree of the network is four and the average number played by each individual is 100. The probability of playing game A is *p* = 0.5. The games were played 30 times with different random numbers, and the corresponding figures were drawn according to the average results of the games. The brown area in the picture demonstrates the weak Parrondo’s paradox area, whereas the green denotes the strong Parrondo’s paradox area.

For each of the above five different behavioral patterns, the influence of the heterogeneity of the degree distribution of networks on the parameter space where the paradox occurs is shown in Fig. 2 through 6 respectively, where the horizontal coordinate denotes the winning probability of branch 1 in game B, *p*_1_,and the vertical coordinate represents the winning probability of branch2 in game B, *p*_2_.The results demonstrate that: (1) any kind of behavior and network structure can produce Parrondo’s paradox; (2) the behavioral patterns have an influence on the parameter space; (3) the higher heterogeneity of the network structure yields a larger region of the parameter space where the paradox occurs.

The simulation results based on the Matthew effect are shown in Fig. 2. Figure 2a shows the paradoxical effect progressively changing from a two-dimensional lattice to a random network. As the rewiring time, *L*, increases, the node degree of the network gradually changes from a *δ*-distribution to a Poisson distribution. The region of parameter space in which the strong paradox occurs (the green region shown in Fig. 2a) progressively increases as well. Figure 2b shows the paradoxical effect progressively changing from a random network to a scale-free network. With the decrease in parameter *α*, the node degree of the network progressively changes from a Poisson distribution to a power-law distribution. The region of parameter space in which the strong paradox occurs (the green region shown in Fig. 2b) is observed to increase as well.

**Figure 3:**
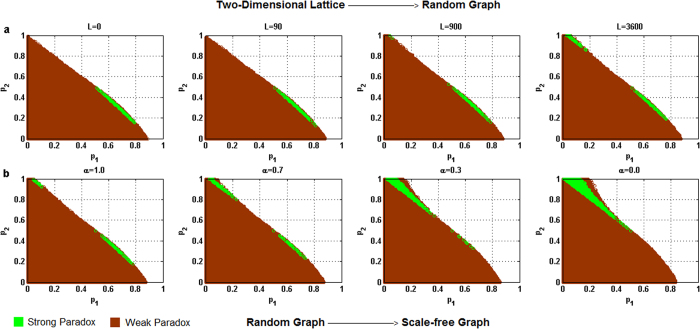
Influence of the heterogeneity of the degree distribution on the paradoxical effect in the harmony pattern. (**a**) The number of rewiring times *L* = 0, 90, 900 and 3,600, which corresponds to the networks changing gradually from a two-dimensional lattice to a random network respectively. (**b**) The parameter α = 1.0, 0.7, 0.3 and 0.0 corresponds to the networks progressively changing from a random network to a scale-free network. The population size *N* is 900. The average degree of the network is four and the average number of played by each individual is 100. The probability of playing game A is *p* = 0.5. The games were played 30 times with different random numbers, and the corresponding figures were drawn according to the average results of the games. The brown area in the picture demonstrates the weak Parrondo’s paradox area, whereas the green denotes the strong Parrondo’s paradox area.

For the harmony-based pattern in Fig. 3, an increment in the heterogeneity of the degree distribution of networks causes the strong paradoxical region (in green) to gradually move towards the upper part of the trapezoidal shape, where the weak paradoxical region (in brown) of the upper part of the trapezoidal shape (which corresponds to the region where *d*^(*A*+*B*)^ > *d*^(*B*)^ > 0 in Fig. 3b) gradually increases, and the weak paradoxical region of the bottom part of the trapezoidal shape (which corresponds to the region where 0 > *d*^(*A*+*B*)^ > *d*^(*B*)^ in Fig. 3b) has no obvious change.

**Figure 4:**
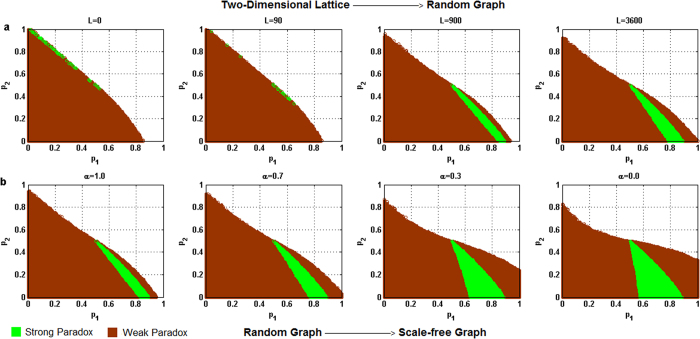
Influence of the heterogeneity of the degree distribution on the paradoxical effect in the cooperative pattern. (**a**) The number of rewiring times *L* = 0, 90, 900 and 3,600, which corresponds to the networks changing gradually from a two-dimensional lattice to a random network respectively. (**b**) The parameter α = 1.0, 0.7, 0.3 and 0.0 corresponds to the networks progressively changing from a random network to a scale-free network. The population size *N* is 900. The average degree of the network is four and the average number played by each individual is 100. The probability of playing game A is *p* = 0.5. The games were played 30 times with different random numbers, and the corresponding figures were drawn according to the average results of the games. The brown area in the picture demonstrates the weak Parrondo’s paradox area, whereas the green denotes the strong Parrondo’s paradox area.

**Figure 5:**
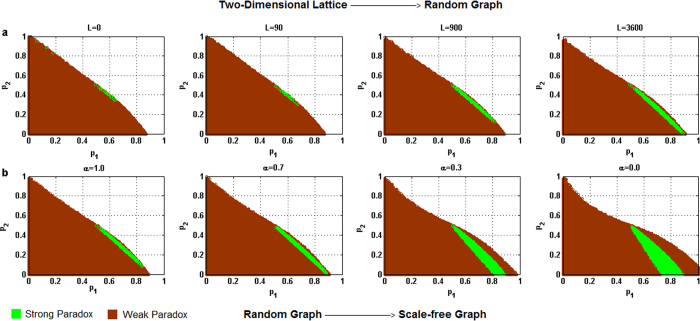
Influence of the heterogeneity of the degree distribution on the paradoxical effect in the PCRC pattern. (**a**) The number of rewiring times *L* = 0, 90, 900 and 3,600, which corresponds to the networks changing gradually from a two-dimensional lattice to a random network respectively. (**b**) The parameter α = 1.0, 0.7, 0.3 and 0.0 corresponds to the networks progressively changing from a random network to a scale-free network. The population size *N* is 900. The average degree of the network is four and the average number played by each individual is 100. The probability of playing game A is *p* = 0.5. The games were played 30 times with different random numbers, and the corresponding figures were drawn according to the average results of the games. The brown area in the picture demonstrates the weak Parrondo’s paradox area, whereas the green denotes the strong Parrondo’s paradox area.

**Figure 6:**
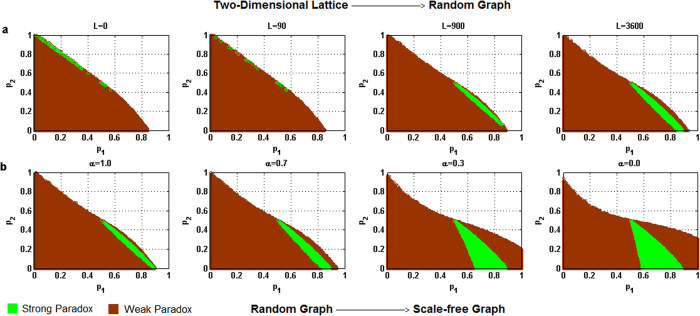
Influence of the heterogeneity of the degree distribution on the paradoxical effect in the random pattern. (**a**) The number of rewiring times *L* = 0, 90, 900 and 3,600, which corresponds to the networks changing gradually from a two-dimensional lattice to a random network respectively. (**b**) The parameter *α* = 1.0, 0.7, 0.3 and 0.0 corresponds to the networks progressively changing from a random network to a scale-free network. The population size *N* is 900. The average degree of the network is four and the average number played by each individual is 100. The probability of playing game A is *p* = 0.5. The games were played 30 times with different random numbers, and the corresponding figures were drawn according to the average results of the games. The brown area in the picture demonstrates the weak Parrondo’s paradox area, whereas the green denotes the strong Parrondo’s paradox area.

Figures 4, 5 and 6 show that with an increase in the heterogeneity of the degree distribution of networks, the trend of changes for the strong and weak paradoxical regions is similar for cooperative, PCRC and random patterns. Figures 4a, 5a and 6a show the results of Parrondo’s paradox progressively changing from a two-dimensional lattice to a random network. With an increase in the heterogeneity of the degree distribution of networks, the strong paradoxical region gradually moves to the bottom part of the trapezoidal shape, where the weak paradoxical region of the upper part of the trapezoidal shape gradually reduces and the weak paradoxical region of the bottom part of the trapezoidal shape gradually increases. Figures 4b, 5b and 6b show the results of Parrondo’s paradox progressively changing from a random network to a scale-free network. As the parameter *α* decreases, the region of the parameter space where the strong paradox (the green region in Figs 4b, 5b and 6b) occurs gradually increases, and the region of the parameter space where the weak paradox occurs (the brown part in Figs 4b, 5b and 6b) is divided into two regions by the green part. The characteristic of the large brown area on the left of the green region is 0 > *d*^(*A*+*B*)^ > *d*^(*B*)^. The characteristic of the small brown area on the right of the green region is *d*^(*A*+*B*)^ > *d*^(*B*)^ > 0. With an increase in the heterogeneity of the network degree distribution, the brown area corresponding to *d*^(*A*+*B*)^ > *d*^(*B*)^ > 0 progressively grows while the brown area corresponding to 0 > *d*^(*A*+*B*)^ > *d*^(*B*)^ progressively reduces.

### The microcosmic mechanism analysis of the paradox

By means of the analysis of the multi-agent Parrondo’s model under five kinds of behavioral patterns, it is known that there are some differences between the strong and the weak paradoxical parameter space when the individuals are located in different network topologies. We take two sets of specific probability parameters, (*p*_1_, *p*_2_), for each of the five behavioral patterns. Here, *p*_1_ denotes the winning probability of branch1 in game B, and *p*_2_ represents the winning probability of branch2 in game B. In one set of probability parameters, (*p*_1_, *p*_2_) satisfies the rules that in the two-dimensional lattice Parrondo’s strong paradox occurs, and in the scale-free network given above, the paradox does not occur. In the other set of probability parameters, (*p*_1_, *p*_2_) satisfies the rules that in the two-dimensional lattice, the paradox does not occur, and in the scale-free network, Parrondo’s strong paradox occurs. Unlike the two-dimensional lattice, the scale-free network has a highly heterogeneous degree distribution. Hence, we hypothesize that the effect of the microcosmic mechanism is the strongest in a scale-free network, but absent in a two-dimensional lattice. The influence of the heterogeneity of degree distributions in the scale-free network on the occurrence (or non-occurrence) of the paradox will be discussed in subsequent sections.

As the behavioral pattern is embodied in the “agitating” mechanism of game A, it only affects the results of the randomized game A + B. Since the microcosmic mechanism is consistent across different behavioral patterns in game B, its results are only affected by the probability parameters. Hence, for the microcosmic mechanism on game B, we will only provide a detailed description for the Matthew behavioral pattern.

#### The Matthew mode

(1) *p*_1_ = 0.175, *p*_2_ = 0.87

For this set of parameters, based on the two-dimensional lattice, the average fitness of the population of game B, *d*^(*B*)^, is 3.37, and the average fitness of the population of the randomized game A + B, *d*^(*A*+*B*)^, is 2.05. Here, *d*^(*B*)^ > *d*^(*A*+*B*)^ > 0. So, the paradox does not occur. Based on the scale-free network, the strong paradox occurs, where the average fitness of the population of game B, *d*^(*B*)^, is −4.56, and the average fitness of the population of the randomized game A + B, *d*^(*A*+*B*)^, is 10.09. The microcosmic mechanism of the strong paradox based on the scale-free network is analyzed for this set of parameters.

**Figure 7:**
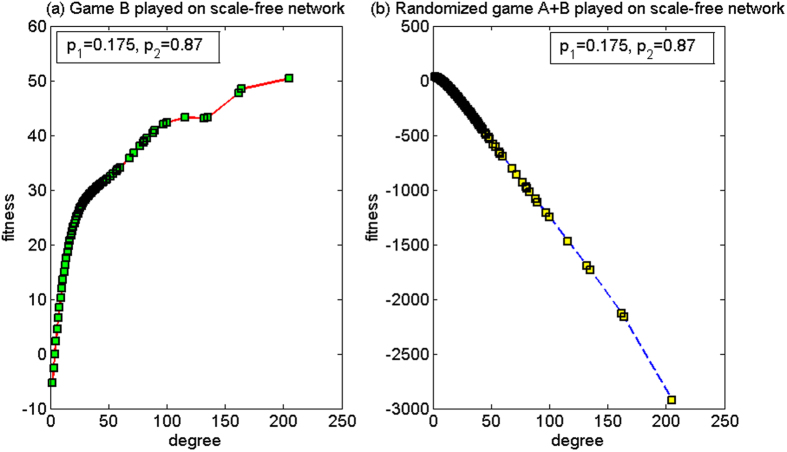
Relationship between the node degree and the fitness of the Matthew mode for the first parameter set (based on the scale-free network). (**a**) When game B is played individually and (**b**) the randomized game A + B. For this set of parameters, the paradox does not occur based on the two-dimensional lattice while the strong paradox occurs based on the scale-free network.The population size *N* is 10,000. The average degree of the network is four and the average number played by each individual is 100. The probability of playing game A is *p* = 0.5. The probabilities of winning in branch 1 and branch 2 of game B are *p*_1_ = 0.175 and *p*_2_ = 0.87 respectively. The figures were based on the average results when the games were played 1,000 times with different random numbers. For the nodes with the same degree, the fitness is averaged from all of these nodes.

Figure 7 shows the relationship between the node degree and the fitness. We observe that, when game B is played individually, a positive relationship exists between the node degree and the fitness. Hence, a node with a larger degree will correspond to a larger fitness. The fitness of nodes with degrees of two and three is negative and the fitness of the other nodes is positive (because the number of nodes with degrees of two and three accounts for 70% of the total amount of nodes in the scale-free network, the average fitness of the population is negative). Similar to the microcosmic mechanism of game B in Fig. 6 of ref. 12, such results occur for parameters *p*_1_ = 0.175 and *p*_2_ = 0.870 because of the asymmetrical construction of game B, as shown in Fig. 1.When the capital of a node is less than or equal to the average capital of all of its neighbors, the neighboring environment of this node becomes unfavorable (branch 1 of game B is played, and the probability of winning is *p*_1_ = 0.175). Otherwise, if the capital of a node is greater than the average capital of all of the neighbors, the neighboring environment of this node is favorable (branch 2 of game B is played and the probability of winning is *p*_2_ = 0.870).In this paper, we assume that the initial capital of all nodes is equal. Thus, the unfavorable branch 1 is played in the beginning, which may result in the decrease of the capital of nodes with smaller degree with a larger probability (because the number of nodes with degrees of two and three accounts for 70% of the total amount of nodes in the scale-free network). At the same time, as the neighboring environment of the nodes with large degree is mainly composed of nodes with smaller degree (nodes with degrees of two and three), the capital of the nodes with smaller degree is small so that the nodes with large degree play the favorable branch 2 with a larger probability, which leads to the increment of the capital of the nodes with large degree. Therefore, the neighboring environment of the small-degree nodes that are connected to these large-degree nodes is further worsened (the increment of the capital of the large-degree nodes causes the average capital of the neighboring environment to produce a comparatively obvious rise). Moreover, this result makes the small-degree nodes play the unfavorable branch 1 with a larger probability. Thus, the capital of the nodes with small degree decreases further. The favorable neighboring environment of the nodes with large degree and the unfavorable neighboring environment of the nodes with small degree are constantly strengthened in the course of play. The final result is that the larger-degree nodes correspond to greater fitness, and the smaller-degree nodes correspond to less fitness (the fitness of the nodes with degrees of two and three is negative, respectively).

On the other hand, when a randomized game A + B is played, we observe that a negative relationship exists between the node degree and the fitness (Fig. 7(b)). The fitness of nodes with node degree less than 10 is positive and the fitness of the other nodes is negative. Although the minimum value of the fitness is approximately −3000, the average fitness of the population is positive as the number of nodes with degrees less than 10 accounts for 94.54% of the total amount of nodes in the scale-free network. The reason for this is the “agitating” role of game A. Half of the time in the randomized game A + B is used to play the zero-sum game A, during which the large-degree nodes will play game A with the small-degree nodes with a higher probability. For the Matthew mode, the system environment is beneficial for the subject when the game starts because of the equal capital of all the individuals at this time. According to Equation (1), at this time when the subject and the object play game A, the probability of winning equals to one. As the number of nodes with degrees less than 10 accounts for 94.54% of the total amount of nodes, game A makes the large-degree nodes (played as the object) pay one unit to the small-degree nodes (played as the subject) with a higher probability. Thus, game A causes the capital of the small-degree nodes (played as the subject) to increase, while the capital of the large-degree nodes (played as the object) to decrease. The capital of the nodes with small degree is now greater. Hence, the nodes with large degree play the unfavorable branch (i.e. branch 1) of game B with a higher probability, leading to the further reduction of the capital of the nodes with large degree. However, the capital of the nodes with larger degree decreases. The nodes with small degree play the favorable branch (i.e. branch 2) of game B with a higher probability, leading to a further increase of the capital of the nodes with small degree. The unfavorable neighboring environment of the large-degree nodes and the favorable neighboring environment of the small-degree nodes are constantly strengthened throughout the course of the game sequence. Under the impetus of the Matthew effect (the phenomenon where “the rich get richer and the poor get poorer”), the large-degree nodes with lower fitness and the small-degree nodes with higher fitness will surface. A drastic difference will appear between the fitness of the nodes with large degree and with small degree. From the viewpoint of the group, the “agitating” role of game A contributes to the increase of the opportunity to play the favorable branch (i.e., branch 2) of game B. The computational results show that the proportion that plays the favorable branch has an increment from 43.48% (when game B is played individually) to 61.28% (when the randomized game A + B is played), which causes the result of the randomized game A + B to be positive. Hence, the “ratcheting” mechanism of game B (several asymmetric branches exist in the structure) and the “agitating” role of game A are key to eliciting Parrondo’s paradox. Simultaneously, the heterogeneity of the degree distributions of networks is advantageous to the play of the “ratcheting” mechanism.

(2) *p*_1_ = 0.53, *p*_2_ = 0.47

For this set of parameters, based on the two-dimensional lattice, the average fitness of the population of game B, *d*^(*B*)^, is −0.04, and the average fitness of the population of the randomized game A + B, *d*^(*A*+*B*)^, is 0.05. Thus, the strong paradox occurs. Based on the scale-free network, the average fitness of the population of game B, *d*^(*B*)^, is 0.16, and the average fitness of the population of the randomized game A + B, *d*^(*A*+*B*)^, is −0.26. Since *d*^(*B*)^ > *d*^(*A*+*B*)^, the paradox does not occur. The microcosmic mechanism of the non-paradox based on the scale-free network is analyzed for this set of parameters below.

**Figure 8:**
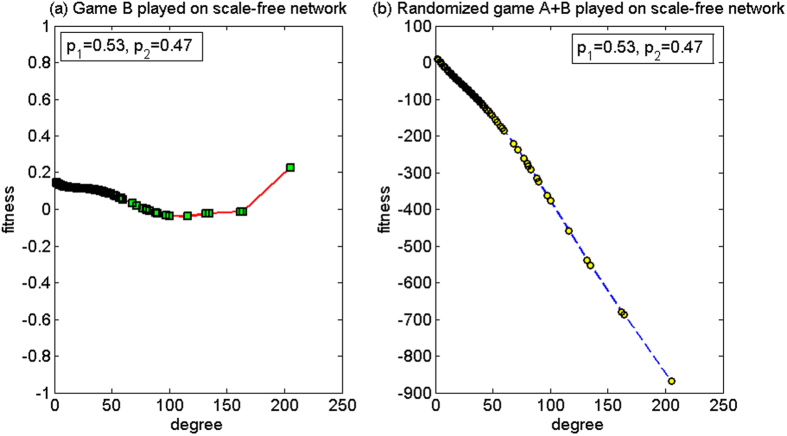
Relationship between the node degree and the fitness of the Matthew mode for the second parameter set (based on the scale-free network). (**a**) When game B is played individually and (**b**) the randomized game A + B. For this set of parameters, the strong paradox occurs based on the two-dimensional lattice while the paradox does not occur based on the scale-free network. The population size *N* is 10,000. The average degree of the network is four and the average number played by each individual is 100. The probability of playing game A is *p* = 0.5. The probabilities of winning in branch 1 and branch 2 of game B are *p*_1_ = 0.53 and *p*_2_ = 0.47 respectively. The figures were based on the average results when the games were played 1,000 times with different random numbers. For the nodes with the same degree, the fitness is averaged from all of these nodes.

Figure 8 shows the relationship between the node degree and the fitness. When game B is played individually, the fitness of the nodes is largely positive, and assumes a uniform, highly localized distribution in a narrow range as shown in Fig. 8(a). The reason for this observation is that, the system environment is advantageous at the beginning of the game (for game B, the favorable branch coincides with a situation in which individuals have equal capital). So regardless of whether a small-degree node or a large-degree node is chosen as an individual to play the game, the probability that the node makes capital gains becomes higher. This increase in the chosen node’s capital improves the neighboring environment of the neighboring nodes, which subsequently raises the chance that a neighboring node will enter the favorable branch (branch 1). The cycle continues, leading to the average population fitness of game B being positive eventually.

Figure 8(b) shows that, when the randomized game A + B is played, an obvious negative relationship exists between the node degree and the fitness. The relationship between the node degree and the fitness is similar to that for the first set of parameters. However, the minimum value (close to −900) is significantly larger than that for the first set of parameters (close to −3000). The difference is that, for the Matthew mode, in the first set of parameters, branch 2 is favorable, and in this second set of parameters, branch 2 is unfavorable. From the viewpoint of the group, the “agitating” role of game A contributes to the increase of the opportunity of the nodes with small degree to play branch 2 of game B. The computational results show that the proportion that plays branch 2 increased from 48.62% (when game B is played individually) to 54.27% (when the randomized game A + B is played). However, note that branch 2 is unfavorable with this second set of parameters, which would lead to a negative outcome for the randomized game A + B. At the same time, nodes with large degree would have greater opportunities to play the favorable branch, which then leads to a higher average fitness than the one with the first set of parameters.

#### The harmony pattern

*p*_1_ = 0.16, *p*_2_ = 0.885

For this set of parameters, based on the two-dimensional lattice, the average fitness of the population of game B, *d*^(*B*)^, is 3.35, and the average fitness of the population of the randomized game A + B, *d*^(*A*+*B*)^, is 0.50. Since *d*^(*B*)^ > *d*^(*A*+*B*)^ > 0, the paradox does not occur. Based on the scale-free network, the strong paradox occurs where the average fitness of the population of game B, *d*^(*B*)^, is −5.43, and the average fitness of the population of the randomized game A + B, *d*^(*A*+*B*)^, is 1.87. The microcosmic mechanism of the strong paradox based on the scale-free network is analyzed for this set of parameters below.

**Figure 9:**
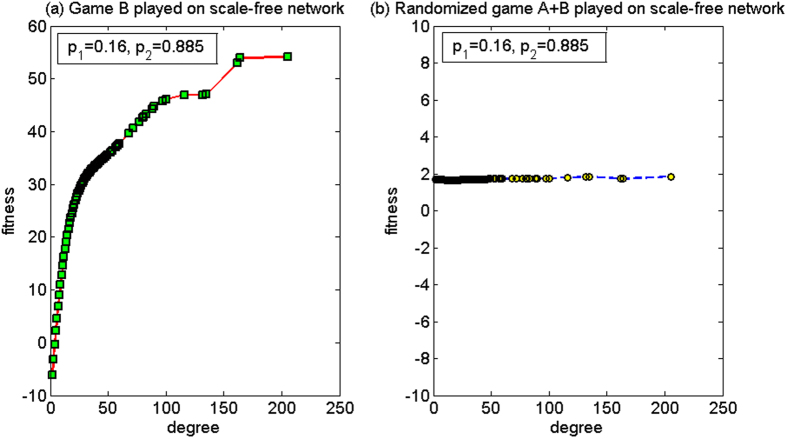
The relationship between the node degree and the fitness of the harmony mode for the first set of parameters (based on the scale-free network). (**a**) When game B is played individually and (**b**) the randomized game A + B. For this set of parameters, the paradox does not occur based on the two-dimensional lattice while the strong paradox occurs based on the scale-free network. The population size *N* is 10,000. The average degree of the network is four and the average number played by each individual is 100. The probability of playing game A is *p* = 0.5. The probabilities of winning in branch 1 and branch 2 of game B are *p*_1_ = 0.16 and *p*_2_ = 0.885, respectively. The figures were based on the average results when the games were played 1,000 times with different random numbers. For the nodes with the same degree, the fitness is averaged from all of these nodes.

Figure 9 shows the relationship between the node degree and the fitness. We observe that when the randomized game A + B is played (see Fig. 9(b)), the node degree has no obvious relationship with the fitness. Basically, the fitness of all the nodes is positive, which makes the average fitness of the population positive. The reason for this result is the “agitating” role of game A. The nodes with large degree and the nodes with small degree will play a zero-sum game among themselves, where nodes with large degree may be chosen more times as an object to play game A with different neighboring nodes with small degree. Furthermore, the game relation is set as the way of harmony, that is, the object with larger capital pays one unit to the subject with smaller or equal capital. Therefore, game A causes the capital of the large-degree nodes in the group to approach the minimum value of the capital in all the neighborhood nodes. The reduction of the capital of the large-degree nodes improves the neighboring environment of the small-degree nodes connected with them. The “agitating” role of game A contributes to the increase in opportunity for the nodes with small degree to play the favorable branch of game B (i.e., branch 2). The computational results show that proportion that plays the favorable branch has an increment from 43.15% (when game B is played separately) to 49.48% (when the randomized game A + B is played). As the entire system environment is largely healthy, the result of the randomized game A + B becomes positive.

(2) *p*_1_ = 0.79, *p*_2_ = 0.155

For this set of parameters, based on the two-dimensional lattice, the average fitness of the population of game B, *d*^(*B*)^, is −0.04, and the average fitness of the population of the randomized game A + B, *d*^(*A*+*B*)^, is 0.14. Thus, the strong paradox occurs. Based on the scale-free network, the average fitness of the population of game B *d*^(*B*)^ is 0.34, and the average fitness of the population of the randomized game A + B, *d*^(*A*+*B*)^, is −1.73. Since *d*^(*B*)^ > *d*^(*A*+*B*)^, the paradox does not occur. The microcosmic mechanism of the non-paradox based on the scale-free network is analyzed for this set of parameters below.

**Figure 10:**
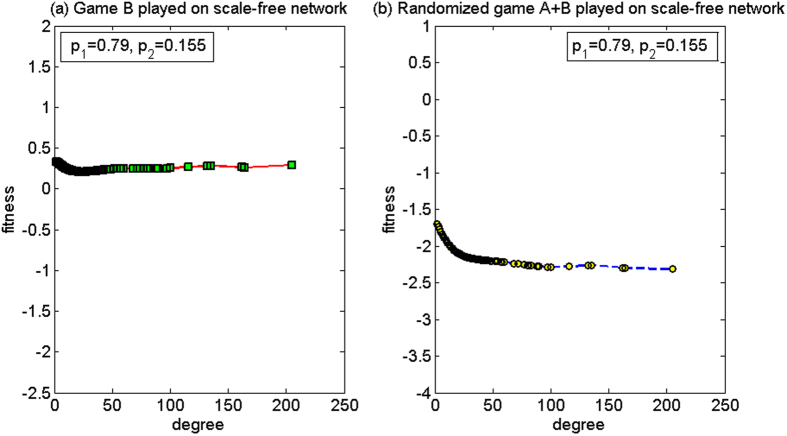
The relationship between the node degree and the fitness of the harmony mode for the second parameter set (based on the scale-free network). (**a**) When game B is played individually and (**b**) the randomized game A + B. For this set of parameters, the strong paradox occurs based on the two-dimensional lattice while the paradox does not occur based on the scale-free network. The population size *N* is 10,000. The average degree of the network is four and the average number played by each individual is 100. The probability of playing game A is *p* = 0.5. The probabilities of winning in branch 1 and branch 2 of game B are *p*_1_ = 0.79 and *p*_2_ = 0.155, respectively. The figures were based on the average results when the games were played 1,000 times with different random numbers. For the nodes with the same degree, the fitness is averaged from all of these nodes.

Figure 10 shows the relationship between the node degree and the fitness. When the randomized game A + B is played (see Fig. 10(b)), the fitness of all the nodes is negative. The fitness of the nodes decreases with the increase of the node degree. This means that, a node with a larger degree corresponds to a lower fitness. This is due to the “agitating” role of game A. Similar to the earlier harmony-based mode in the first set of parameters, game A causes the capital of the large-degree nodes in the group to approach the minimum value of the capital in all the neighborhood nodes. The difference for the harmony mode is that, for the first group of parameters, branch 2 is the favorable branch, and for this second group of parameters, branch 2 is the unfavorable branch. The neighboring environment of the small-degree nodes connected with the large-degree nodes has deteriorated with the reduction of the capital of the large-degree nodes (for the first set of parameters, as branch 2 is favorable, the increment of the capital of the large-degree nodes improves the neighboring environment of the small-degree nodes connected with them). This raises the opportunity for the nodes with small degree to play the unfavorable branch 2 of game B. The computational results show that the proportion that plays the unfavorable branch increased from 45.40% when game B is played individually to 48.39% when the randomized game A + B is played, which leads to a reduction of the capital of the small-degree nodes. The reduction of the capital for the small-degree nodes causes further decline of the capital for the large-degree nodes through game A. This cycle continues, eventually leadings to the fitness of all nodes being negative.

#### The cooperative pattern

(1) *p*_1_ = 0.09, *p*_2_ = 0.92

For this set of parameters, based on the two-dimensional lattice, the average fitness of the population of game B, *d*^(*B*)^, is −0.17, and the average fitness of the population of the randomized game A + B, *d*^(*A*+*B*)^, is 0.05. Thus, the strong paradox occurs. Based on the scale-free network, the paradox will not occur, where the average fitness of the population of game B *d*^(*B*)^ is −12.56, and the average fitness of the population of the randomized game A + B, *d*^(*A*+*B*)^, is −23.63. The microcosmic mechanism of the non-paradox based on the scale-free network is analyzed for this set of parameters below.

**Figure 11:**
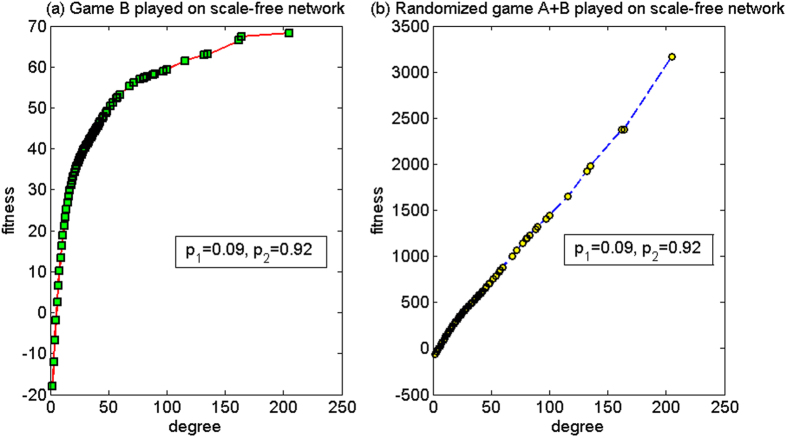
The relationship between the node degree and the fitness of the cooperative pattern for the first set of parameters (based on the scale-free network). (**a**) When game B is played individually and (**b**) the randomized game A + B. For this set of parameters, the strong paradox occurs based on the two-dimensional lattice while the paradox does not occur based on the scale-free network. The population size *N* is 10,000. The average degree of the network is four and the average number played by each individual is 100. The probability of playing game A is *p* = 0.5. The probabilities of winning in branch 1 and branch 2 of game B are *p*_1_ = 0.09 and *p*_2_ = 0.92, respectively. The figures were based on the average results when the games were played 1,000 times with different random numbers. For the nodes with the same degree, the fitness is averaged from all of these nodes.

Figure 11 shows the relationship between the node degree and the fitness. When the randomized game A + B is played, Fig. 11(b) reveals that a positive relationship exists between the node degree and the fitness, where the fitness of the small-degree nodes of two, three and four is negative and the fitness of other nodes is positive. Compared with game B, node degrees of two, three and four lose more and the fitness is smaller. Take a node with a degree of two as an example. The fitness for game B played individually is −18.5068 and the fitness for the randomized game A + B is −68.0092. Furthermore, the positive fitness is much higher than the one for game B played individually. The maximum value of the fitness is approximately 3200. However, as the number of nodes with degrees of two, three and four accounts for 80% of the total amount of nodes in the scale-free network, the average fitness of the population is negative. The reason for this result is due to the “agitating” role of game A. Half of the time in the randomized game A + B is used to play the zero-sum game A, where the nodes with large degree and the nodes with small degree will play game A among themselves with a higher probability. Furthermore, the game relation is set as way of cooperation. For the cooperative pattern, the subject pays one unit to the object for free. As the number of the nodes with small degrees of two, three, and four accounts for 80% of the total amount of nodes, game A makes the nodes with small degree (played as the subject) pay one unit for free to the nodes with large degree (played as the subject) with a higher probability. Thus, game A causes the capital of the nodes with small degree (played as the subject) to reduce and the capital of the nodes with large degree (played as the object) to increase. The favorable neighboring environment of the nodes with large degree and the unfavorable neighboring environment of the nodes with small degree are constantly strengthened in the course of play. A drastic difference eventually appears between the capital of the nodes with large degree and nodes with small degree. From the viewpoint of the group, for nodes with small degree, the opportunity in playing the favorable branch 2 of game B has a great reduction by the “agitating” role of game A. The computational results show that the proportion that plays the favorable branch has a decline from 41.83% (when game B is played individually) to 20.92% (when the randomized game A + B is played). Thus, the average fitness of the randomized game A + B is further reduced, compared to that of game B.

(2) *p*_1_ = 0.835, *p*_2_ = 0.06

For this set of parameters, based on the two-dimensional lattice, the average fitness of the population of game B, *d*^(*B*)^, is −2.78, and the average fitness of the population of the randomized game A + B, *d*^(*A*+*B*)^, is −3.92. Since *d*^(*B*)^ > *d*^(*A*+*B*)^, the paradox does not occur. Based on the scale-free network, the average fitness of the population of game B, *d*^(*B*)^, is −2.11 and the average fitness of the population of the randomized game A + B, *d*^(*A*+*B*)^, is 24.68. Since *d*^(*A*+*B*)^ > 0 and *d*^(*B*)^ < 0, the strong paradox occurs. The microcosmic mechanism of the strong paradox based on the scale-free network is analyzed for this set of parameters below.

**Figure 12:**
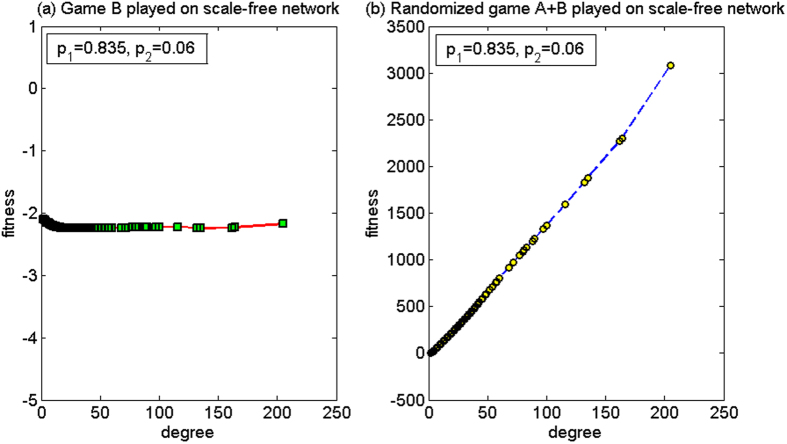
The relationship between the node degree and the fitness of the cooperative mode for the second parameter set (based on the scale-free network). (**a**) When game B is played individually and (**b**) the randomized game A + B. For this set of parameters, the paradox does not occur based on the two-dimensional lattice while the strong paradox occurs based on the scale-free network. The population size *N* is 10,000. The average degree of the network is four and the average number played by each individual is 100. The probability of playing game A is *p* = 0.5. The probabilities of winning in branch 1 and branch 2 of game B are *p*_1_ = 0.835 and *p*_2_ = 0.06, respectively. The figures were based on the average results when the games were played 1,000 times with different random numbers. For the nodes with the same degree, the fitness is averaged from all of these nodes.

Figure 12 shows the relationship between the node degree and the fitness. Since it is not more advantageous for this set of parameters (*p*_1_ = 0.835, *p*_2_ = 0.06) than in the second set of parameters for the Matthew mode (*p*_1_ = 0.53, *p*_2_ = 0.47) and for the harmony mode (*p*_1_ = 0.79, *p*_2_ = 0.155), the average fitness of the population for game B is negative. However, the microcosmic mechanism is consistent with the above two behavior modes.

When the randomized game A + B is played, Fig. 12(b) reveals that a positive relationship exists between the node degree and the fitness, which is similar to the first set of parameters as in the above cooperation-based mode. However, the difference for cooperation is that, for the first group of parameters, branch 1 is the unfavorable branch, and for this second group of parameters, branch 1 is the favorable branch. Although the nodes with small degree pay one unit for free to the nodes with large degree with a higher probability, the increase in the capital of the large-degree nodes improves the neighboring environment of the small-degree nodes connected with them. For the first set of parameters, branch 1 is unfavorable. The neighboring environment of the small-degree nodes connected with the large-degree nodes has deteriorated with the increase in the capital of the large-degree nodes. For the nodes with small degree, this result causes the increase in the opportunity to play the favorable branch 1 of game B. The computational results show that the proportion that plays the unfavorable branch has an increment from 55.42% (when game B is played individually) to 88.62% (when the randomized game A + B is played). The average fitness of the small-degree nodes of two, three and four in this set has an increment respectively, compared with that in the first set of parameters. The average fitness of the node with a degree of two rises from −68.0092 to −2.6943, while the average fitness of the nodes with degrees of three and four turns from negative to positive. As only the fitness of the nodes with a small degree of two (accounting for 50.23% of the population in the scale-free network) is negative, and the fitness of other nodes is positive, the population average fitness of the randomized game A + B becomes positive.

#### The PCRC pattern

(1) *p*_1_ = 0.13, *p*_2_ = 0.885

For this set of parameters, based on the two-dimensional lattice, the average fitness of the population of game B *d*^(*B*)^ is −0.04, and the average fitness of the population of the randomized game A + B, *d*^(*A*+*B*)^, is 0.04. Since *d*^(*A*+*B*)^ > 0 and *d*^(*B*)^ < 0, hence the strong paradox occurs. Based on the scale-free network, the paradox does not occur, where the average fitness of the population of game B, *d*^(*B*)^, is −9.58, and the average fitness of the population of the randomized game A + B, *d*^(*A*+*B*)^, is −14.89. The microcosmic mechanism of the non-paradox based on the scale-free network is analyzed for this set of parameters below.

**Figure 13:**
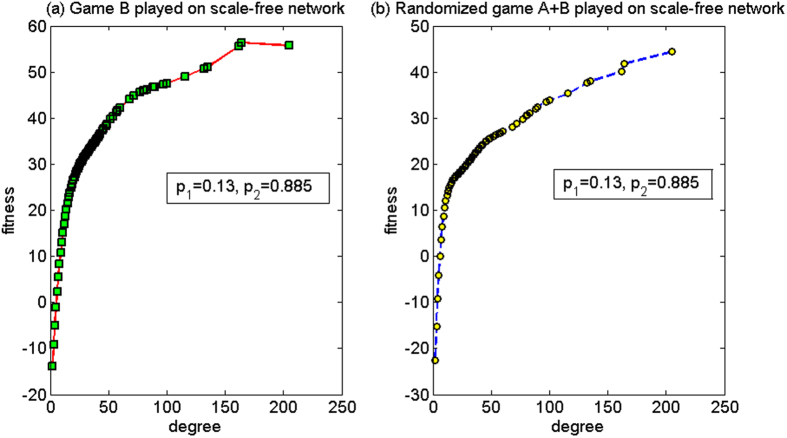
The relationship between the node degree and the fitness of the PCRC mode for the first set of parameters (based on the scale-free network). (**a**) When game B is played individually and (**b**) the randomized game A + B. For this set of parameters, the strong paradox occurs based on the two-dimensional lattice while the paradox does not occur based on the scale-free network. The population size *N* is 10,000. The average degree of the network is four and the average number played by each individual is 100. The probability of playing game A is *p* = 0.5. The probabilities of winning in branch 1 and branch 2 of game B are *p*_1_ = 0.13 and *p*_2_ = 0.885, respectively. The figures were based on the average results when the games were played 1,000 times with different random numbers. For the nodes with the same degree, the fitness is averaged from all of these nodes.

Figure 13 shows the relationship between the node degree and the fitness. When the randomized game A + B is played (see Fig. 13(b)), the fitness of nodes with degrees of two, three, four and five is negative and the fitness of the other nodes is positive (because the number of nodes with degrees of two, three, four and five accounts for 85.47% of the total amount of nodes in the scale-free network, the average fitness of the population is negative). For the randomized game A + B, a certain positive relationship exists between the node degree and the fitness. This relationship is different from that in the harmony mode in the first set of parameters (there is no obvious relationship between the fitness and the node degree) and that for the cooperation mode in the first set of parameters (there is an obvious positive relation between the fitness and the node degree and the fitness has a significant increment). This is because game A behaves according to PCRC. If the capital of the subject is less than or equal to that of the object, the subject and the object will compete; otherwise, the subject and the object will cooperate, that is the subject pays one unit to the object for free. As the nodes with small degree (the nodes with degrees of two, three, four and five accounting for 85.47% of the total amount of nodes) play as the subject, while the nodes with large degree play as the object with a higher probability; when the capital of the small-degree nodes is less than or equal to that of the large-degree nodes, competition results in the fitness between the small-degree and the large-degree nodes being uniform.

When the capital of the small-degree nodes is greater than that of the large-degree nodes, cooperation makes the small-degree node pay one unit to the large-degree nodes. Therefore, game A with the PCRC mode will cause the capital of the large-degree nodes in the group to tend towards being the largest in the capital of all the neighborhood nodes. Thus, a certain positive relationship exists between the fitness and the node degree. It is neither like the first set of parameters for the cooperation mode where significant difference exists between them, nor in the case of the first set of parameters for the harmony mode where there is no significant relationship between them. This positive relationship renders the nodes with large degree to be in a favorable neighboring environment and nodes with small degree to be in an unfavorable neighboring environment. From the viewpoint of the group, the “agitating” role of game A leads to the decrease in the opportunity to play the favorable branch 2 of game B for nodes with small degree. The computational results reveal that the proportion that plays the favorable branch will decline from 42.66% (when game B is played individually) to 29.28% (when the randomized game A + B is played). Therefore, the average fitness of the randomized game A + B is less than that of game B being played individually.

(2) *p*_1_ = 0.82, *p*_2_ = 0.095

For this set of parameters, based on the two-dimensional lattice, the average fitness of the population of game B *d*^(*B*)^ is −1.61, and the average fitness of the population of the randomized game A + B, *d*^(*A*+*B*)^, is −1.88. Since *d*^(*B*)^ > *d*^(*A*+*B*)^, hence the paradox does not occur. Based on the scale-free network, the average fitness of the population of game B, *d*^(*B*)^, is −1.09, and the average fitness of the population of the randomized game A + B, *d*^(*A*+*B*)^, is 9.97. Since *d*^(*A*+*B*)^ > 0 and *d*^(*B*)^ < 0, hence the strong paradox occurs. The microcosmic mechanism of the strong paradox based on the scale-free network is analyzed for this set of parameters below.

**Figure 14:**
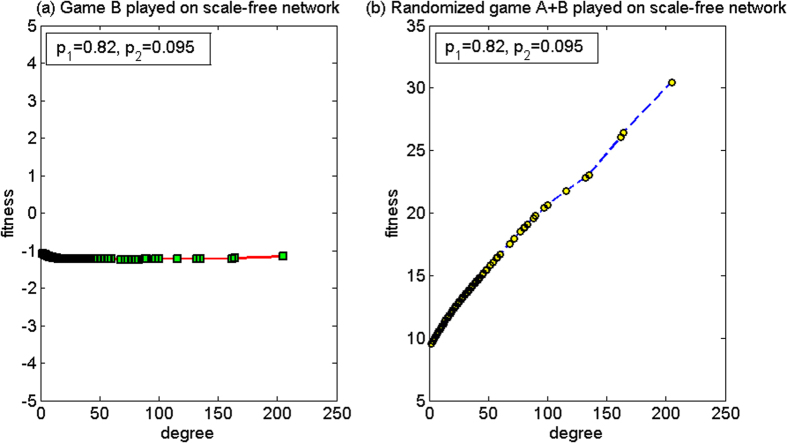
The relationship between the node degree and the fitness of the PCRC mode for the second parameter set (based on the scale-free network). (**a**) When game B is played individually and (**b**) the randomized game A + B. For this set of parameters, the paradox does not occur based on the two-dimensional lattice while the strong paradox occurs based on the scale-free network. The population size *N* is 10,000. The average degree of the network is four and the average number played by each individual is 100. The probability of playing game A is *p* = 0.5. The probabilities of winning in branch 1 and branch 2 of game B are *p*_1_ = 0.82 and *p*_2_ = 0.095, respectively. The figures were based on the average results when the games were played 1,000 times with different random numbers. For the nodes with the same degree, the fitness is averaged from all of these nodes.

Figure 14 shows the relationship between the node degree and the fitness. When the randomized game A + B is played (see Fig. 14(b)), the node degree has a positive relationship with the fitness. Furthermore, the fitness of all the nodes is positive. This is because, for this group of parameters, branch 1 is favorable, while for the first set of parameters of the above PCRC mode, branch 1 is unfavorable. Similar to the first set of parameters, game A with the PCRC mode causes the capital of the large-degree nodes in the group to tend towards being the largest in the capital of all the neighborhood nodes. Thus, a certain positive relationship exists between the fitness and the node degree. The main difference from the first set of parameters is that, this positive relationship renders small-degree nodes to be in a favorable neighboring environment. Thus, the opportunity for the nodes with small degree to play the favorable branch 1 of game B increases. The computational results reveal that the proportion that plays the favorable branch has an increment from 55.11% (when game B is played individually) to 69.62% (when the randomized game A + B is played). Therefore, the result of the randomized game A + B is positive.

#### The random pattern

(1) *p*_1_ = 0.255, *p*_2_ = 0.755

**Figure 15:**
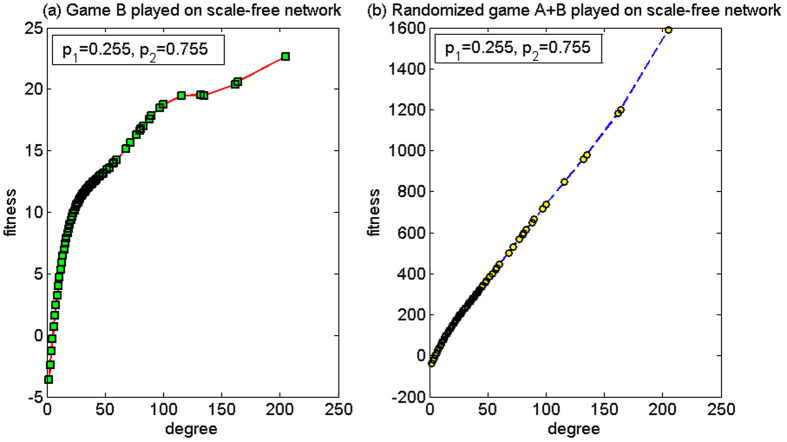
The relationship between the node degree and the fitness of the random mode for the first set of parameters (based on the scale-free network). (**a**) When game B is played individually and (**b**) the randomized game A + B. For this set of parameters, the strong paradox occurs based on the two-dimensional lattice while the paradox does not occur based on the scale-free network. The population size *N* is 10,000. The average degree of the network is four and the average number played by each individual is 100. The probability of playing game A is *p* = 0.5. The probabilities of winning in branch 1 and branch 2 of game B are *p*_1_ = 0.255 and *p*_2_ = 0.755, respectively. The figures were based on the average results when the games were played 1,000 times with different random numbers. For the nodes with the same degree, the fitness is averaged from all of these nodes.

The case for this set of parameters is similar to the first set of parameters in the cooperative mode, where the strong paradox occurs in the two-dimensional lattice and the paradox does not occur in the scale-free network. The only difference is that, the fitness of the nodes with large degree under the random behavior is smaller than that of the nodes with large degree under the cooperative pattern, which is approximately 1/2 (comparing Fig. 11(b) with Fig. 15(b)). This is because, in the random mode, the subject chooses the cooperative strategy with probability of 0.5 and the competitive strategy with probability of 0.5. When a cooperative strategy is chosen, the result is similar to that of the cooperative behavior. Game A widens gap in the fitness of the nodes with large degree and that of the nodes with small degree. However, when a competitive strategy is chosen, small-degree nodes may win the large-degree nodes, thereby closing the gap between them. Since half of the time of game A is used for the competitive strategy, the difference of the fitness among the nodes is not as significant as the first set of parameters for the cooperation mode, as shown in Fig. 15(b).

(2)*p*_1_ = 0.835, *p*_2_ = 0.06

**Figure 16:**
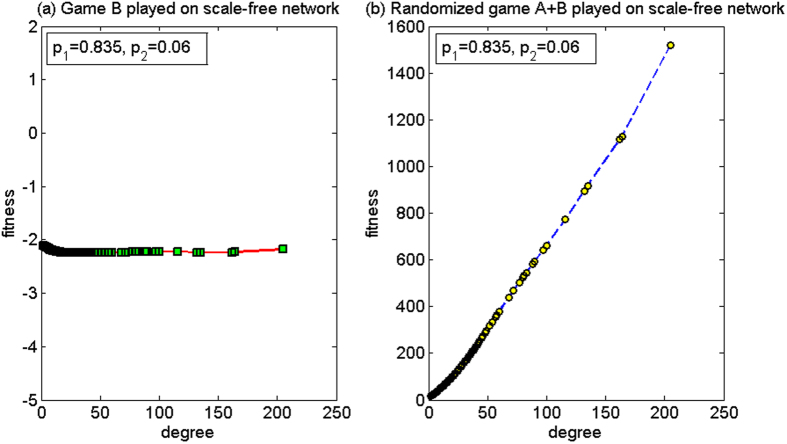
The relationship between the node degree and the fitness of the random mode for the second parameter set (based on the scale-free network). (**a**) When game B is played individually and (**b**) the randomized game A + B. For this set of parameters, the paradox does not occur based on the two-dimensional lattice while the strong paradox occurs based on the scale-free network. The population size *N* is 10,000. The average degree of the network is four and the average number played by each individual is 100. The probability of playing game A is *p* = 0.5. The probabilities of winning in branch 1 and branch 2 of game B are *p*_1_ = 0.835 and *p*_2_ = 0.06, respectively. The figures were based on the average results when the games were played 1,000 times with different random numbers. For the nodes with the same degree, the fitness is averaged from all of these nodes.

The case for this set of parameters is similar to the second set of parameters in the cooperative mode, where the paradox does not occur in the two-dimensional lattice and the strong paradox occurs in the scale-free network. The only difference is that the fitness of the nodes with large degree under the random behavior is smaller than that of the nodes with large degree under the cooperative behavior, which is approximately 1/2 (comparing Fig. 12(b) with Fig. 16(b)). The reason for this result is consistent with the reason of the first set of parameters in the random mode.

### Limitations and Future Work

The current study is based on analyzing the gradual change in the parameter space where Parrondo’s paradox occurs, when the network changes from a two-dimensional lattice to a random network and from a random network to a scale-free network. It may be possible to find analytical representations, like those carried out in refs 16, 17, 18, 19, which might help in understanding the five different behavioral patterns better. Whether such analytical functions exist is a subject for future work.

## Conclusions

According to the multi-agent Parrondo’s model based on complex networks in ref. 12, the current study proposed for the individual interactions in game A to be characterized into five types of behavioral patterns: the Matthew effect, harmony, cooperation, poor-competition-rich-cooperation (PCRC) and the random pattern. The relationship between the parameter space and the heterogeneity of the degree distribution of the network was also analyzed. The simulation results reveal that: 1) any kind of behavioral pattern and network structure can elicit the Parrondo’s paradox; 2) a higher heterogeneity yields a larger region of the parameter space where the paradox occurs. The heterogeneity of the degree distributions of networks is conducive to the play of the “ratcheting” mechanism, which may also be the reason for the high heterogeneity of most networks in reality; 3) The behavioral patterns have an influence on the parameter space of the paradox. The size of the region of the paradoxical parameter space for the cooperative behavior has the most obvious increase as the heterogeneity of degree distribution of networks increase. Based on the scale-free network, the region of the paradoxical parameter space is the largest for the cooperative behavior in game A. Therefore, the heterogeneity of the network has a positive impact on the adaptability of the cooperative pattern.

For each of the five kinds of behavioral patterns, we investigated how two sets of probability parameters alter the relationship between node degree and fitness. In one set of probability parameters, branch 1 of game B is unfavorable (e.g. *p*_1_ is small), and in the other set of probability parameters branch 1 of game B is favorable (e.g. *p*_1_ is large). Common interaction mechanisms among the asymmetric structure of game B, the “agitating” role of game A in different behavioral patterns and network topology structures are revealed.

Finally, for the Matthew effect, even though the analysis of the parameter space where the paradox occurs appears to be similar to that of the competitive behavior^12^, the juxtaposition of Fig. 7 in this paper and Fig. 6 of ref. 12 reveals that, based on the scale-free network, the microcosmic mechanism of the randomized game A + B where the strong paradox occurs is completely different.

## Additional Information

**How to cite this article**: Ye, Y. *et al*. Effects of behavioral patterns and network topology structures on Parrondo’s paradox. *Sci. Rep*. **6**, 37028; doi: 10.1038/srep37028 (2016).

**Publisher’s note**: Springer Nature remains neutral with regard to jurisdictional claims in published maps and institutional affiliations.
